# Selection of solvent for extraction of antioxidant components from *Cynanchum auriculatum*,* Cynanchum bungei*, and *Cynanchum wilfordii* roots

**DOI:** 10.1002/fsn3.967

**Published:** 2019-02-20

**Authors:** Cheng‐Dong Wu, Ming Zhang, Ming‐Tao He, Min‐Feng Gu, Mei Lin, Gen Zhang

**Affiliations:** ^1^ Xinyang Agricultural Experiment Station of Yancheng City Yancheng China; ^2^ Shenzhen GenProMetab Biotechnology Company Limited Shenzhen China

**Keywords:** antioxidant activity, *Cynanchum*, dietary supplement, extraction

## Abstract

In east Asia, “Baishouwu” has been used as an herbal drug and functional dietary supplement for hundreds of years. Actually, “Baishouwu” is the common name of the roots of *Cynanchum auriculatum*,* Cynanchum bungei*, and *Cynanchum wilfordii*. In the present study, roots of these three specie were extracted and then fractionated using petroleum ether (PE), dichloromethane (DCM), ethyl acetate (EA), and water. DPPH scavenging experiments revealed high antioxidant activity of DCM and EA fractions of *C. bungei* and the EA fraction of *C. wilfordii*. Treatments with these three fractions significantly reduced malondialdehyde content in heat‐stressed *Daphnia magna*, validating in vivo antioxidant activity. Gas chromatography–mass spectrometer (GC‐MS) analyses demonstrated that the chemical components of fractions extracted from *C. bungei*,* C. bungei*, and *C. wilfordii* were different. Further determination of total phenol and total flavonoids contents showed that DCM and EA fractions of *C. bungei* and EA fraction of *C. wilfordii* had much higher contents of total phenol and total flavonoids, which might be the reason to explain their strong antioxidant activity. Overall, the present study suggested that these three plants have different chemical components and biological activities. They could not be used as the same drug.

## 1 INTRODUCTION

“Baishouwu” is a famous tonic herbal drug in traditional Chinese medicine and also named “Baekshuoh” in Korean Pharmacopoeia (Jiang et al., 2011). As traditionally suggested, “Baishouwu” has functions in enriching vital essence and enhancing immunity (Shan et al., 2006). Modern pharmacological researches showed that “Baishouwu” functions in antitumor (Shan et al., 2005), clearance of free radicals (Song & Ding, 1997), enhancement of immunity (Gu, Gong, Tao, Liu, & Zhao, 1987), reduction in serum cholesterol level (Niu, Ye, & Wang, 1988), and protection of gastro (Shan et al., 2006). Due to these beneficial functions, “Baishouwu” is not only used as herbal drugs or sources of modern drugs, but also applied as healthcare food or functional food supplements.

“Baishouwu” is not from a single plant species. It is the appellative name of dried root tubers from three Asclepiadaceae plants, including *Cynanchum auriculatum* Royle ex Wight, *Cynanchum bungei* Decne, and *Cynanchum wilfordii* Maxim. The chemical components and the pharmaceutical functions of these three species might be different. For example, the ultraviolet–visible spectrum (UV‐Vis), Fourier transform infrared spectroscopy (FT‐IR), and high‐performance liquid chromatography (HPLC) analysis showed significant differences between the extracts of *C. auriculatum* and *C. bungei* (Liu et al., 2013). More detailed, a bunch of glucosides (Gan, Xiang, Ma, & Hu,  2010; Li, Kadota, Kawata, Hattori, & Namba, 1993), two natural acetophenone derivatives including 2,5‐dihydroxyacetophenone (2,5‐DHAP) and 2,6‐DHAP (Ding, Chang, Shen, & Tai, 2011; Liu et al., 2008), a pair of novel epimers (Li et al. 2018), displaying inhibitory effects on proliferation of B and T lymphocytes, murine tyrosinase, and tumorigenesis, respectively, were purified from *C. bungei*. Similarly, the same and novel C21‐steroidal glycosides were also detected in *C. auriculatum* and *C. wilfordii* (Li et al., 2008; Lu, Xiong, Hong, Guang, & Zhi, 2011; Xiang, Ma, & Hu, 2010; Ye, Wang, Yang, & Zhang, 2013; Zhang, Ye, Shen, & Liang, 2000). Besides, novel compounds, such as Wilfoside K1N inhibiting angiogenesis (Kim et al., 2005), cynandione A protecting from cytotoxicity (Lee et al., 2015), and amino‐substituted p‐benzoquinone (Yeo & Kim, 1997), were isolated from roots of *C. wilfordii*. Obviously, due to the different chemical components among *C. auriculatum*,* C. bungei*, and *C. wilfordii*, the healthcare effects of these three species should be different. Misuse of these three plants might reveal no effects or even trigger adverse effects to human body. To develop healthcare food and functional dietary supplements from “Baishouwu,” it is necessary to investigate the bioactivity of three species.

Antioxidant activity is an important function of dietary supplements (Wasek et al., 2015). It was claimed that *C. wilfordii* (Lee, Kim, & Le, 2001), *C. auriculatum* (Chen et al., 2013), and *C. bungei* (Chen, Chen, Ma, Zhang, & Zhou, 2017) contained antioxidant compounds. The relative antioxidant activity of *C. wilfordii*,* C. auriculatum*, and *C. bungei* has not been compared. Moreover, solvent was proved to significantly affect the effectiveness of antioxidant compound extraction from plants (Spigno, Tramelli, & De Faveri, 2007; Sultana, Anwar, & Ashraf, 2009; Turkmen, Sari, & Velioglu, 2006). Due to the variation of chemical components in *C. auriculatum*,* C. bungei*, and *C. wilfordii*, the best solvent for the extraction of antioxidant compounds should be investigated. These pieces of information would promote the development of antioxidant dietary supplements from “Baishouwu” extracts.

In the present study, to compare the antioxidant activity of three “Baishouwu” species and to search the most efficient solvent for antioxidant compound extraction, roots of *C. auriculatum*,* C. bungei*, and *C. wilfordii* were extracted using ethanol solution and then fractionated using petroleum ether (PE), dichloromethane (DCM), ethyl acetate (EA), and water. The antioxidant activity of these fractions was tested by calculating the scavenging rate of 1,1‐diphenyl‐2‐picrylhydrazyl (DPPH) radicals and monitoring changes of antioxidant indices is heat‐stressed *Daphnia magna*. Gas chromatography–mass spectrometer (GC‐MS) was applied to analyze the chemical components of these fractions, and contents of total phenol and total flavonoids were determined. These results might be useful for the development of functional dietary supplements from “Baishouwu.”

## 2 MATERIALS AND METHODS

### 2.1 Materials

Dry roots of *C. auriculatum*,* C. bungei* (produced in Binhai, Jiangsu Province, China), and *C. wilfordii* (produced in Tai'an, Shandong Province, China) were purchased from the local planting bases. To identify species, genomic DNA was extracted from these samples using BioFastSpin plant genomic DNA extraction kit (Bioer, Hangzhou, China). Ribosomal DNA internal transcribed spacer (ITS) seuqences were amplified using BioReady Hot Start Taq (Bioer, Hangzhou, China) and primers of 5'‐GTCGAATTCGTAGGTGAACCTGCGGAAGGATCA‐3' and 5'‐CCTGCAGTCGACAKATGCTTAARTTCAGCRGG‐3', sequenced and then blasted against Genbank database. The final results confirmed the species of these three samples. The roots were dried at 60°C for 24 hr to completely remove moisture and then sealed in plastic bags before analysis. All reagents used in the present study were HPLC grade.

### 2.2 Chemical extraction

All the three samples were ground into powers using an electronic grinder. Then, approximately 1 kg of each species was weighed. For chemical extraction, samples were soaked in 4 L of 80% ethanol for 1 hr with sonication. After centrifugation at 5,980 *g* for 5 min, the supernatant was collected and the pallets were further extracted in 4 L of 60% ethanol for 1 hr with sonication. This step using 60% ethanol was repeated once. All the supernatants were pooled, filtered through a filter paper, and concentrated by rotary evaporation (Heidolph Hei‐VAP, Germany).

The extracts were dispersed in 300 ml of Milli‐Q water and sequentially extracted using 400 ml of petroleum ether (PE), 400 ml of dichloromethane (DCM), and 400 ml of ethyl acetate (EA). Extraction with each solvent was repeated for three times, and the two layers were separated using separating funnels. Extracts obtained by the same solvent were pooled and dried by rotary evaporation (Heidolph Hei‐VAP). Finally, four factions were collected, including PE fraction, DCM fraction, EA fraction, and water fraction. All fractions were dried, precisely weighed, and then dissolved in DMSO to prepare 50 mg/ml stock solution.

### 2.3 Determination of DPPH radical scavenging activity

The DPPH radical scavenging activity was determined following Wong, Chai, and Hoo (2012) with modifications. To prepare DPPH solution, 8 mg of DPPH was dissolved in 50 ml of methanol and then further diluted by adding 50 ml of Milli‐Q water. The butyl hydroxy anisd (BHA) solution was prepared by dissolving 1 mg of BHA in 40 μl of methanol. These two solutions were freshly prepared just before use.

The tests of DPPH radical scavenging activity were performed in 24‐well plates. In each well, 1 ml of DPPH solution and 1 μl of sample solution were mixed and then placed at room temperature in dark for 30 min. The final concentrations of extracts included 50, 25, 20, 12.5, 10, and 6.25 mg/L by diluting the stock solution using DMSO. Next, the absorbance at 517 nm was determined using a microplate reader (FlexStation3, Molecular Devices, USA). The sample solution was replaced by DMSO as the negative control. BHA solution was used instead of sample solution as the positive control. The rate of scavenging DPPH radicals was calculated using the following formula.

$$\text{DPPH radical scavenging rate}=[\left({\text{A}}_{0}-{\text{A}}_{s}\right)/{\text{A}}_{0}]\;\times \;100\%$$

where A_s_ represents the absorbance of reaction containing sample solution, and A_0_ is the absorbance in the negative control.

Each test was repeated three times and the EC_50_ was calculated using the Probit method in SPSS 23.

### 2.4 Effects of extracts on antioxidant indices in *Daphnia magna*

To validate antioxidant activity detected by DPPH radical scavenging experiments, *D. magna* individuals were treated with heat shock and extract fractions. Changes of antioxidant indices were compared. Briefly, *D. magna* was maintained at 20°C using EPA medium and fed 5.0 × 10^5^ cells/ml *Scenedesmus obliquus*. Experiments were carried out in 50‐ml glass beakers. In each beaker, 150 individuals were placed and then treated with 10 mg/L EA and DCM fraction of *C. bungei* extract and 10 mg/L EA fraction of *C. wilfordii* extract. The culture temperature increased from 20 to 30°C to trigger heat stress. After incubation at 30°C for 24 hr, all animals were harvested to detect activities of superoxide dismutase (SOD, Dong, Zhao, Huang, & Wen, 2011), peroxidase (POD, Upadhyaya, Sankhla, Davis, Sankhla, & Smith, 1985), catalase (CAT, Johansson & Borg, 1988), and content of malondialdehyde (MDA, Draper & Hadley, 1990) as described previously. Concentration of total protein was assayed using a Bradford protein assay kit (Tiangen, Beijing, China). One unit of SOD activity was defined as the amount of enzyme required for 1 mg of tissue proteins in 1 ml of reaction mixture to achieve SOD inhibition rate of 50%. One unit of CAT activity was defined as 1 mg of tissue proteins consumed 1 μmol H_2_O_2_ in 1 s. One unit of POD activity was defined as increase in absorbance at 420 nm per 1 mg of tissue proteins in 1 min at 37°C. Enzyme activities were expressed as U/mg proteins, and content of MDA was expressed as nmol/mg proteins. Each assay was repeated three times.

### 2.5 Gas chromatography–mass spectrometer (GC‐MS) analysis

For analysis of chemical components, 1 μl of each sample was injected into the GC‐MS (Agilent7890A‐5975C) equipped with the HP‐5 column (30 m × 0.32 mm × 0.25 μm) with the split ratio of 10:1. Helium was used as the carrier gas with the flow rate of 1.5 ml/min, and the temperature of the injection port and the detector was set at 220°C. All data were obtained by collecting the full‐scan mass spectra. The spectra information was aligned with the database to identify chemicals.

### 2.6 Determination of total phenol (TP)

Contents of TP were determined using a plant total phenol test kit (Solarbio, Beijing, China) following the manufacturer's protocol. Briefly, 50 μl of sample solution was mixed with 250 μl of Folin–Ciocalteu reagent for 2 min at room temperature and then was neutralized using 250 μl of sodium carbonate solution (7.5%, w/v). The reaction mixture was incubated at room temperature for 10 min with intermittent shaking for color development. Afterward, the reaction solution was diluted with 450 μl of distilled water and absorbance at 760 nm was determined using a UV2800 UV‐Vis spectrophotometer (Unico, Shanghai, China). Gallic acid was used to prepare the standard curve. Each assay was repeated three times.

### 2.7 Determination of total flavonoids (TF)

Contents of TF were determined using a plant total flavonoids test kit (Solarbio, Beijing, China) following the manufacturer's protocol. Briefly, 0.2 ml of sample solution was reacted with 0.3 ml of ethanol, 0.05 ml of 10% aluminum chloride, 0.05 ml of 1 M potassium acetate, and 0.4 ml of distilled water at 37°C for 45 min. Absorbance of the reaction mixture was measured at 450 nm using a UV2800 UV‐Vis spectrophotometer (Unico, Shanghai, China). Gallic acid was used to prepare the standard curve. Each assay was repeated three times.

## 3 RESULTS AND DISCUSSION

The antioxidant activity of PE, DCM, EA, and water fractions extracted from *C. auriculatum*,* C. bungei*, and *C. wilfordii* was tested using the DPPH radical scavenging tests. At the concentration of 50 mg/L, all fractions of *C. auriculatum*, PE and water fractions of *C. bungei* as well as PE, DCM, and water fractions of *C. wilfordii* did not reveal obvious DPPH radical scavenging activity. DCM and EA fractions of *C*. *bungei* as well as EA fraction of *C. wilfordii* showed significantly lower absorbance at 517 nm in comparison  to the blank control (DMSO), thus these three fractions were considered as antioxidant fractions.

**Figure 1:**
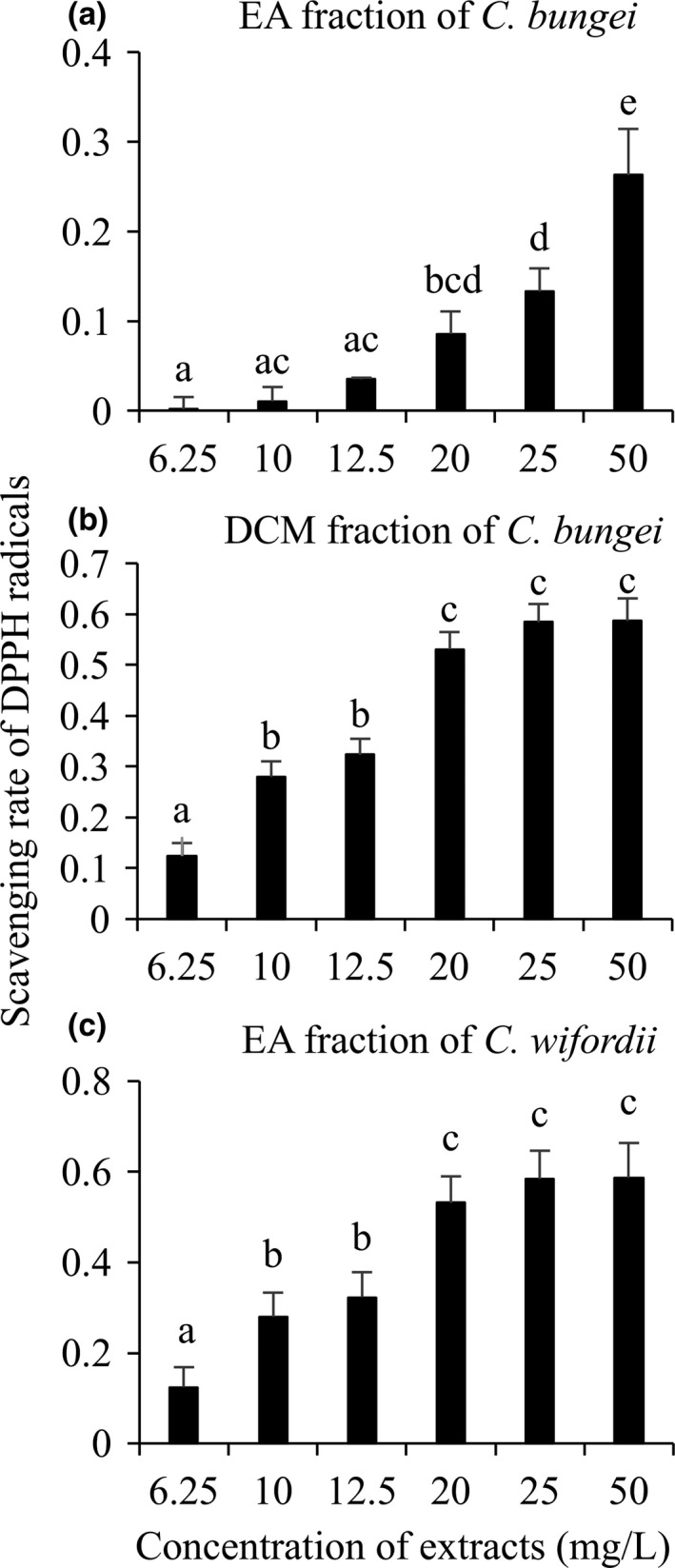
DPPH radical scavenging activities of dichloromethane (DCM) and ethyl acetate (EA) fractions of *Cynanchum bungei* and *C. wilfordii* (mean ± *SD*)

The fractions with preliminary antioxidant activity were further tested with more concentrations. In the range of tested concentrations (6.25–50 mg/L), all treatments significantly scavenged DPPH radicals. Along with increasing concentration of extracts, the scavenging rate of EA fraction of *C. bungei* increased significantly. The scavenging rate of DCM fraction of *C. bungei* and EA fraction of *C. wifordii* also increased with elevating concentration. However, when the concentration arrived 20 mg/L, the scavenging rate of DPPH radicals became stable (Figure 1). The EC_50_ values of DPPH radical scavenging activity were calculated. The lowest EC_50_ was observed in the EA fraction of *C. wilfordii*, equal to 30.74 mg/L, following by the EA fraction and the DCM fraction of *C. bungei*. However, based on the tested range of concentrations (0–50 mg/L), the EC_50_ of other fractions of *C. bungei* and all fractions of *C. bungei* could not be calculated (Table 1).

**Table 1:** EC_50_ values of DPPH radical scavenging activity of *C. auriculatum*,* C. bungei*, and *C. wilfordii* extracts

Species	Fraction	EC_50_
*C. bungei*	Petroleum ether	Not available
	Dichloromethane	69.12 mg/L
	Ethyl acetate	46.81 mg/L
	Water	Not available
*C. auriculatum*	Petroleum ether	Not available
	Dichloromethane	Not available
	Ethyl acetate	Not available
	Water	Not available
*C. wilfordii*	Petroleum ether	Not available
	Dichloromethane	Not available
	Ethyl acetate	30.74 mg/L
	Water	Not available

Heat stress effectively induced reactive oxygen species (ROS) in *D. magna* (Becker, Brinkmann, Zeis, & Paul, 2012; Klumpen et al., 2017) and peroxidation of membrane lipids, resulting in elevation of MDA content. Under stress environments, *D. magna* generally increases activities of SOD, POD, and CAT to resist harmful effects of ROS (Kim, Kim, Kim, Won, & Lee, 2018). Treatment with *C. bungei* extract decreased MDA content and increased SOD activity in mouse brain, displaying antioxidant and neuroprotective effects (Chen et al., 2017). In the present study, partially similar results were displayed. Compared with the control, treatments with EA, DCM fractions of *C. bungei* and EA fraction of *C. wilfordii* significantly decreased MDA content but did not affect activities of CAT, SOD, and POD (Table 2), suggesting that heat stress‐induced membrane lipid peroxidation might be directly suppressed by these fractions, which was consistent with the results of DPPH assays. Overall, these results suggested that *C. bungei* and *C. wilfordii* had antioxidant activity and might be applied as healthcare food and functional food supplements. For manufacturing the functional food supplements, *C. bungei* could be extracted using DCM and/or EA, but only EA could be used to extract the antioxidant components from *C. wilfordii*. Zhang, Tao, Cui, and Ao (2006) optimized the extraction procedure of antioxidant compounds from *C. bungei* and found that 75% ethanol solution was the best solvent. The present study further found that EA and/or DCM might be used to further purify antioxidant compounds from the ethanol extracts.

**Table 2:** Changes of antioxidant indices in *Daphnia magna* treated with 10 mg/L ethyl acetate and dichloromethane fractions of *C. bungei* and *C. wilfordii* extracts (mean ± *SD*)

Species/fraction	CAT (U/mg prot)	POD (U/mg prot)	SOD (U/mg prot)	MDA (nmol/mg prot)
Control	4.87 ± 1.60	29.8 ± 5.8	16.4 ± 1.6	40.3 ± 6.2
*Cb*/EA	7.15 ± 1.91	40.5 ± 25.3	18.1 ± 3.0	26.8 ± 2.3^a^
*Cb*/DCM	5.10 ± 0.28	28.1 ± 11.3	15.2 ± 3.5	21.9 ± 3.7^a^
*Cw*/EA	5.55 ± 1.06	29.2 ± 1.5	16.8 ± 0.7	20.2 ± 2.4^a^

*Note*

*Cb*:* C. bungei*;* Cw*:* C. wilfordii*; DCM: dichloromethane; EA: ethyl acetate.

a Significantly different from the control.

Gas chromatography–mass spectrometer analysis detected a bunch of chemicals from DCM and EA fractions of extracts. However, the chemical components of these three plants were quite different. Only thiocyanic acid, ethyl ester, triphenyl phosphate, dibutyl phthalate, 9,12‐octadecadienoic acid (Z, Z)‐ and methyl ester were detected in the DCM fraction of all the three tested samples. Thiocyanic acid, ethyl ester, dibutyl phthalate, benzenamine, N, N‐dimethyl‐4‐(phenylazo)‐, hexadecanoic acid, and ethyl ester were shared by EA fractions of the three species (Supporting Information Tables S1 and S2). These results suggested that *C*. *auriculatum*,* C. bungei*, and *C. wilfordii* contained different chemicals, which might show different effects to human bodies. When “Baishouwu” was applied as healthcare food or functional dietary supplement, these three species could not replace mutually.

We tried to search the chemicals functioning as antioxidant in the present study. DCM fraction of *C. bungei* revealed antioxidant activity, but DCM fractions of the other two species did not. GC‐MS results of DCM fractions were compared. Two compounds, isopropyl alcohol and 2‐methyl‐2‐Propanol, were observed in the DCM fraction of *C. bungei* but not in those of *C. auriculatum* and *C. wilfordii*. Obviously, these two chemicals should not have any antioxidant activity. To find out the antioxidant compounds in the DCM fraction of *C. bungei*, more investigations should be carried out.

Only triphenyl phosphate and 9‐octadecenoic acid (Z)‐, methyl ester were detected in EA fractions of both *C*. *bungei* and *C. wilfordii* but not in *C. auriculatum*. However, it has been reported that triphenyl phosphate was toxic to *D. magna* and zebrafish (Isales et al., 2015; Lin, 2008), which should not be the reason for the antioxidant activity in EA fractions of *C*. *bungei* and *C. wilfordii*. 9‐octadecenoic acid (Z)‐, methyl ester is easy to be autoxidized (Deatherage & Mattill, 1939). It was possible that this compound absorbed radicals produced by DPPH and appeared as a possible reason for the antioxidant activity of EA extracts. However, more studies should be conducted to confirm this viewpoint.

The GC‐MS method is usually used to analyze volatile constituents. However, constituents of DCM and EA fractions are generally not volatile. Phenol and flavonoids are two typical groups of compounds in plants, revealing DPPH radical scavenging activity and antioxidant activity (Adebayo, Arsad, & Samian, 2018; Zhang, Yang, & Zhou, 2018). In addition to GC‐MS analyses, contents of TP and TF were detected. The results showed that DCM and EA fractions of *C*. *bungei* and EA fraction of *C. wilfordii* revealed 3–75 times higher levels of TF and TP than other fractions (Table 3).

**Table 3:** Contents of total phenol and total flavonoids in *C. auriculatum*,* C. bungei*, and *C. wilfordii* extracts (mean ± *SD*)

Species	Fraction	Total phenol (mg/g)	Total flavonoids (mg/g)
*C. bungei*	Petroleum ether	2.94 ± 0.04	13.43 ± 2.19
	Dichloromethane	10.18 ± 0.28	49.58 ± 0.00
	Ethyl acetate	42.38 ± 1.54	96.69 ± 0.88
	Water	2.94 ± 0.28	9.56 ± 1.10
*C. auriculatum*	Petroleum ether	20.78 ± 2.44	88.06 ± 10.59
	Dichloromethane	121.60 ± 2.56	404.42 ± 6.57
	Ethyl acetate	157.14 ± 7.48	438.50 ± 24.10
	Water	11.73 ± 0.93	26.34 ± 0.00
*C. wilfordii*	Petroleum ether	7.50 ± 0.16	66.11 ± 2.19
	Dichloromethane	35.72 ± 4.39	257.47 ± 6.94
	Ethyl acetate	222.67 ± 7.44	237.07 ± 4.38
	Water	35.72 ± 0.65	83.41 ± 3.29

Overall, these results demonstrated that DCM and EA fractions of *C*. *bungei* and EA fraction of *C. wilfordii* showed significant in vitro and in vivo antioxidant activity probably through phenol and/or flavonoids compounds.

## CONFLICT OF INTEREST

The authors declare that they do not have any conflict of interest.

## ETHICAL STATEMENTS

This study does not involve any human or mammal testing. Experiments on *Daphnia magna* did not require any approval in P. R. China.
