# Supplementary material for: Selection of solvent for extraction of antioxidant components from Cynanchum auriculatum, Cynanchum bungei, and Cynanchum wilfordii roots
Source: Food Sci Nutr. 2019 Feb 20;7(4):1337–43. doi: 10.1002/fsn3.967 (PMC6475734; doi:10.1002/fsn3.967)
Supplement: Supplementary file 1 [file FSN3-7-1337-s001.docx]

**Table S1. GC-MS analysis of dichloromethane fractions extracted from *Cynanchum auriculatum*, *C. bungei* and *C. wilfordii*. ND: not detected.**

| CAS number | Chemical name | Ratio of peak area (%) | | |
| --- | --- | --- | --- | --- |
|  |  | *C. bungei* | *C. auriculatum* | *C. wilfordii* |
| 542-90-5 | Thiocyanic acid, ethyl ester | 40.30 | 19.79 | 17.81 |
| 10049-04-4 | Chlorine dioxide | 17.19 | 6.13 | ND |
| 50-18-0 | 2-[Bis(2-chloroethylamino)]-tetrahydro-2H-1,3,2-oxazaphosphorine-2-oxide | 0.21 | 0.04 | ND |
| 115-86-6 | Triphenyl phosphate | 1.11 | 0.27 | 0.44 |
| 67-63-0 | Isopropyl Alcohol | 0.58 | ND | ND |
| 75-65-0 | 2-Propanol, 2-methyl- | 0.08 | ND | ND |
| 36653-82-4 | 1-Hexadecanol | 2.72 | 1.27 | ND |
| 930-55-2 | Pyrrolidine, 1-nitroso- | 0.51 | ND | ND |
| 62-75-9 | N-Nitrosodimethylamine | 0.23 | ND | 0.09 |
| 117-81-7 | Bis(2-ethylhexyl) phthalate | 3.43 | ND | 1.82 |
| 84-74-2 | Dibutyl phthalate | 1.69 | 0.05 | 0.04 |
| 628-97-7) | Hexadecanoic acid, ethyl ester | 13.04 | ND | ND |
| 142-50-7 | 1,6,10-Dodecatrien-3-ol, 3,7,11-trimethyl-, [S-(Z)]- | 15.55 | ND | ND |
| 107-19-7 | 2-Propyn-1-ol | 0.02 | ND | ND |
| 112-63-0 | 9,12-Octadecadienoic acid (Z,Z)-, methyl ester | 3.34 | 2.24 | 0.93 |
| 111-76-2 | Ethanol, 2-butoxy- | ND | 5.50 | ND |
| 67-68-5 | Dimethyl Sulfoxide | ND | 1.75 | ND |
| 112-12-9 | 2-Undecanone | ND | 0.25 | ND |
| 10102-43-9 | Nitric oxide | ND | 0.04 | 2.03 |
| 108-68-9 | Phenol, 3,5-dimethyl- | ND | 0.05 | ND |
| 60-11-7 | Benzenamine, N,N-dimethyl-4-(phenylazo)- | ND | 0.03 | 0.04 |
| 463-58-1 | Carbonyl sulfide | ND | 0.11 | ND |
| 1634-04-4 | Propane, 2-methoxy-2-methyl- | ND | 0.39 | 0.79 |
| 97-54-1 | Phenol, 2-methoxy-4-(1-propenyl)- | ND | 0.37 | ND |
| 498-02-2 | Ethanone, 1-(4-hydroxy-3-methoxyphenyl)- | ND | 0.89 | ND |
| 510-15-6 | Chlorobenzilate | ND | 0.29 | ND |
| 612-00-0 | Benzene, 1,1'-ethylidenebis- | ND | 0.48 | ND |
| 534-52-1 | Phenol, 2-methyl-4,6-dinitro- | ND | 0.05 | ND |
| 91-16-7 | Benzene, 1,2-dimethoxy- | ND | 0.51 | ND |
| 306-08-1 | Benzeneacetic acid, 4-hydroxy-3-methoxy- | ND | 1.19 | ND |
| 486-25-9 | 9H-Fluoren-9-one | ND | 0.04 | ND |
| 55-91-4 | Isoflurophate | ND | 1.28 | ND |
| 118-93-4 | Ethanone, 1-(2-hydroxyphenyl)- | ND | 5.72 | ND |
| 1420-07-1 | Phenol, 2-(1,1-dimethylethyl)-4,6-dinitro- | ND | 0.01 | ND |
| 21564-17-0 | Thiocyanic acid, (2-benzothiazolylthio)methyl ester | ND | 0.23 | ND |
| 40321-76-4 | 1,2,3,7,8-Pentachlorodibenzodioxin | ND | 0.16 | 0.25 |
| 2478-38-8 | Ethanone, 1-(4-hydroxy-3,5-dimethoxyphenyl)- | ND | 0.53 | ND |
| 460-19-5 | Ethanedinitrile | ND | 0.22 | 0.26 |
| 134-96-3 | Benzaldehyde, 4-hydroxy-3,5-dimethoxy- | ND | 0.10 | ND |
| 208-96-8 | Acenaphthylene | ND | 1.96 | ND |
| 58-08-2 | Caffeine | ND | 0.79 | ND |
| 506-77-4 | Cyanogen chloride | ND | 0.09 | 0.07 |
| 614-96-0 | 1H-Indole, 5-methyl- | ND | 0.04 | ND |
| 86-74-8 | Carbazole | ND | 0.50 | ND |
| 121-14-2 | Benzene, 1-methyl-2,4-dinitro- | ND | 0.14 | ND |
| 79-46-9 | Propane, 2-nitro- | ND | 1.07 | ND |
| 124-10-7 | Methyl tetradecanoate | ND | 0.55 | ND |
| 629-62-9 | Pentadecane | ND | 0.90 | ND |
| 124-04-9 | Hexanedioic acid | ND | 2.76 | ND |
| 475-20- 7 | 1,4-Methanoazulene, decahydro-4,8,8-trimethyl-9-methylene-, [1S-(1.alpha.,3a.be | ND | 0.64 | ND |
| 95-80-7 | 1,3-Benzenediamine, 4-methyl- | ND | 0.02 | ND |
| 112-39-0 | Hexadecanoic acid, methyl ester | ND | 0.61 | 0.58 |
| 80-62-6 | 2-Propenoic acid, 2-methyl-, methyl ester | ND | 0.07 | ND |
| 57-10-3 | n-Hexadecanoic acid | ND | 25.74 | 39.52 |
| 628-97-7 | Hexadecanoic acid, ethyl ester | ND | 4.12 | 7.42 |
| 110-86-1 | Pyridine | ND | 1.42 | ND |
| 110-83-8 | Cyclohexene | ND | 0.09 | ND |
| 76-22-2 | Camphor | ND | 1.06 | ND |
| 128-37-0 | Butylated Hydroxytoluene | ND | 0.13 | 0.16 |
| 842-07-9 | 2-Naphthalenol, 1-(phenylazo)- | ND | 0.01 | 0.06 |
| 108-50-9 | Pyrazine, 2,6-dimethyl- | ND | 0.03 | ND |
| 540-73-8 | Hydrazine, 1,2-dimethyl- | ND | 0.76 | ND |
| 79-92-5 | Camphene | ND | 0.45 | 1.10 |
| 100-51-6 | Benzyl Alcohol | ND | 1.73 | ND |
| 112-62-9 | 9-Octadecenoic acid (Z)-, methyl ester | ND | 3.79 | 2.23 |
| 1120-21-4 | Undecane | ND | 0.44 | ND |
| 226-36-8 | Dibenz(a,h)acridine | ND | 0.12 | ND |
| 298-04-4 | Disulfoton | ND | ND | 1.17 |
| 116-06-3 | Aldicarb | ND | ND | 2.34 |
| 115-86-6 | Triphenyl phosphate | 1.11 | 0.27 | 0.44 |
| 838-88-0 | Benzenamine, 4,4'-methylenebis[2-methyl- | ND | ND | 0.02 |
| 60-11-7 | Benzenamine, N,N-dimethyl-4-(phenylazo)- | ND | 0.03 | 0.04 |
| 1634-04-4 | Propane, 2-methoxy-2-methyl- | ND | 0.39 | 0.79 |
| 7637-07-2 | Boron trifluoride | ND | ND | 0.03 |
| 62-75-9 | N-Nitrosodimethylamine | 0.23 | ND | 0.09 |
| 80-56-8 | alpha.-Pinene | ND | ND | 0.03 |
| 40321-76-4 | 1,2,3,7,8-Pentachlorodibenzodioxin | ND | 0.16 | 0.25 |
| 67-72-1 | Ethane, hexachloro- | ND | ND | 0.19 |
| 3442-78-2 | Pyrene, 2-methyl- | ND | ND | 0.05 |
| 535-77-3 | Benzene, 1-methyl-3-(1-methylethyl)- | ND | ND | 0.37 |
| 141-93-5 | Benzene, 1,3-diethyl- (ID#:141-93-5) | ND | ND | 0.72 |
| 70-69-9 | 1-Propanone, 1-(4-aminophenyl)- | ND | ND | 0.08 |
| 84-74-2 | Dibutyl phthalate | 1.69 | 0.05 | 0.04 |
| 626-38-0 | 2-Pentanol, acetate | ND | ND | 0.19 |
| 112-39-0 | Hexadecanoic acid, methyl ester | ND | 0.61 | 0.58 |
| 1836-75-5 | Benzene, 2,4-dichloro-1-(4-nitrophenoxy)- | ND | ND | 2.57 |
| 117-81-7 | Bis(2-ethylhexyl) phthalate | 3.43 | ND | 1.82 |
| 57-10-3 | n-Hexadecanoic acid | ND | 25.74 | 39.52 |
| 628-97-7 | Hexadecanoic acid, ethyl ester | ND | 4.12 | 7.42 |
| 124-06-1 | Tetradecanoic acid, ethyl ester | ND | ND | 0.07 |
| 128-37-0 | Butylated Hydroxytoluene | ND | 0.13 | 0.16 |
| 112-42-5 | 1-Undecanol | ND | ND | 16.21 |
| 112-63-0 | 9,12-Octadecadienoic acid (Z,Z)-, methyl ester | 3.34 | 2.24 | 0.93 |
| 79-92-5 | Camphene | ND | 0.45 | 1.10 |
| 112-62-9 | 9-Octadecenoic acid (Z)-, methyl ester | ND | 3.79 | 2.23 |
| 10102-43-9 | Nitric oxide | ND | 0.04 | 2.03 |
| 101-05-3 | Anilazine | ND | ND | 0.15 |
| 506-77-4 | Cyanogen chloride | ND | 0.09 | 0.07 |
| 67-66-3 | Trichloromethane | ND | ND | 0.18 |

**Table S2. GC-MS analysis of ethyl acetate fractions extracted from *Cynanchum auriculatum*, *C. bungei* and *C. wilfordii*. ND: not detected.**

| CAS number | Chemical name | Ratio of peak area | | |
| --- | --- | --- | --- | --- |
|  |  | *C. bungei* | *C. auriculatum* | *C. wilfordii* |
| 67-68-5 | Dimethyl Sulfoxide | 0.55 | ND | ND |
| 542-90-5 | Thiocyanic acid, ethyl ester | 0.54 | 38.52 | 40.85 |
| 110-43-0 | 2-Heptanone | 0.04 | ND | ND |
| 7637-07-2 | Boron trifluoride | 0.00 | ND | ND |
| 115-86-6 | Triphenyl phosphate | 0.02 | ND | 0.46 |
| 75-65-0 | 2-Propanol, 2-methyl- | 0.04 | ND | ND |
| 67-63-0 | Isopropyl Alcohol | 0.00 | ND | ND |
| 99-04-7 | Benzoic acid, 3-methyl- | 0.00 | ND | ND |
| 79-19-6 | Hydrazinecarbothioamide | 0.02 | ND | ND |
| 36653-82-4 | 1-Hexadecanol | 2.61 | ND | ND |
| 112-62-9 | 9-Octadecenoic acid (Z)-, methyl ester | 0.03 | ND | 0.68 |
| 950-37-8 | Methidathion | 0.01 | ND | ND |
| 62-75-9 | N-Nitrosodimethylamine | 0.00 | 0.14 | ND |
| 84-74-2 | Dibutyl phthalate | 0.27 | 2.87 | 0.14 |
| 60-11-7 | Benzenamine, N,N-dimethyl-4-(phenylazo)- | 0.00 | 0.08 | 0.02 |
| 10102-43-9 | Nitric oxide | 0.07 | ND | ND |
| 610-72-0 | Benzoic acid, 2,5-dimethyl- | 95.46 | ND | ND |
| 628-97-7 | Hexadecanoic acid, ethyl ester | 0.05 | 0.30 | 4.69 |
| 75-15-0 | Carbon disulfide | 0.00 | 0.11 | ND |
| 57-97-6 | Benz[a]anthracene, 7,12-dimethyl- | 0.00 | ND | ND |
| 110-83-8 | Cyclohexene | 0.07 | ND | ND |
| 76-22-2 | Camphor | 0.01 | ND | 5.06 |
| 132-32-1 | 9H-Carbazol-3-amine, 9-ethyl- | 0.08 | ND | ND |
| 1897-45-6 | Tetrachloroisophthalonitrile | 0.01 | ND | ND |
| 675-14-9 | 1,3,5-Triazine, 2,4,6-trifluoro- | 0.07 | ND | ND |
| 353-50-4 | Carbonic difluoride | ND | 11.00 | ND |
| 7446-09-5 | Sulfur dioxide | ND | 0.58 | ND |
| 1634-04-4 | Propane, 2-methoxy-2-methyl- | ND | 0.03 | 0.03 |
| 14667-55-1 | Pyrazine, trimethyl- | ND | 0.23 | ND |
| 504-24-5 | 4-Pyridinamine | ND | 0.78 | 0.85 |
| 120-71-8 | Benzenamine, 2-methoxy-5-methyl- | ND | 22.84 | 1.66 |
| 7784-42-1 | Arsine | ND | 10.50 | ND |
| 106-44-5 | Phenol, 4-methyl- | ND | 3.91 | ND |
| 118-93-4 | Ethanone, 1-(2-hydroxyphenyl)- | ND | 3.27 | ND |
| 463-58-1 | Carbonyl sulfide | ND | 0.59 | ND |
| 498-02-2 | Ethanone, 1-(4-hydroxy-3-methoxyphenyl)- | ND | 1.34 | ND |
| 86-74-8 | Carbazole | ND | 2.08 | ND |
| 112-63-0 | 9,12-Octadecadienoic acid (Z,Z)-, methyl ester | ND | 0.83 | 2.56 |
| 116-06-3 | Aldicarb | ND | ND | 4.98 |
| 2847-72-5 | Decane, 4-methyl- | ND | ND | 15.41 |
| 821-55-6 | 2-Nonanone | ND | ND | 1.87 |
| 106-96-7 | 1-Propyne, 3-bromo- | ND | ND | 0.20 |
| 84-66-2 | Diethyl Phthalate | ND | ND | 1.35 |
| 460-19-5 | Ethanedinitrile | ND | ND | 0.01 |
| 40321-76-4 | 1,2,3,7,8-Pentachlorodibenzodioxin | ND | ND | 0.19 |
| 535-77-3 | Benzene, 1-methyl-3-(1-methylethyl)- | ND | ND | 0.16 |
| 70-69-9 | 1-Propanone, 1-(4-aminophenyl)- | ND | ND | 0.05 |
| 112-39-0 | Hexadecanoic acid, methyl ester | ND | ND | 0.25 |
| 117-81-7 | Bis(2-ethylhexyl) phthalate | ND | ND | 1.46 |
| 57-10-3 | n-Hexadecanoic acid | ND | ND | 15.42 |
| 1490-04-6 | Menthol | ND | ND | 1.66 |
